# Rare Cause of Tension Pneumothorax: Hydatid Disease of Lung: A Case Report

**DOI:** 10.31729/jnma.4693

**Published:** 2020-04-30

**Authors:** Achyut Bhakta Acharya, Narendra Bhatta, Deebya Raj Mishra, Avatar Verma, Rejina Shahi

**Affiliations:** 1 Department of Pulmonary, Critical Care and Sleep Medicine, B. P. Koirala Institute of Health Sciences, Dharan, Nepal

**Keywords:** *echinococcosis*, *pneumothorax*, *rare cause*, *rupture*

## Abstract

Lung is the second most common site of hydatid disease, after liver. Pleural involvement of hydatid disease can occur, and usually follows the rupture of a pulmonary or hepatic hydatid cyst into the pleural space. When a patient presents with tension pneumothorax, zoonotic infections, especially hydatid disease of the lung, also has to be considered especially in areas with high burden of the disease. We report a 31 years male patient presenting with tension pneumothorax due to rupture of hydatid cyst of lung.

## INTRODUCTION

Hydatid disease is caused by larvae of Echinococcus. In the life cycle of E. granulosus, humans sometimes become accidental intermediate hosts. The hydatid disease is endemic in some Mediterranean countries, Middle East, South America and South Africa and Oceania.^1^ Hydatid disease is seen in any age and sex, although it is more common in those aged 20-40 years.^2,3^ Clinical symptoms depend on the location, number, and size of the cysts. The liver is the most common site of infection followed by the lung in 10% to 30% of cases, and other sites as spleen, kidney, brain, and in bone collectively about 10% cases. In lungs approximately 60% are located in the lower lobes, and a predilection for the right lung is seen in 56% cases.^4^ Thoracic hydatidosis that are extrapulmonary are rare.^5,6^ Pleural involvement of hydatid disease can occur, and usually follows the rupture of a pulmonary or hepatic hydatid cyst into the pleural space. Pleural involvement can be in the form of pleural effusion or rarely, pneumothorax. We present a case of pneumothorax secondary to rupture of hydatid cyst in the lungs.

## CASE REPORT

Thirty one years old man,non-smoker, occasional alcohol consumer, without any illness in the past presented to emergency department with increased shortness of breath associated with left sided pleuritic chest pain for six days with blood tinged sputum for one day without history of fever. Clinically his trachea was shifted to right side with reduced air entry on left side with hyper-resonant percussion note. His oxygen saturation was 90% in room air, was tachypneic with respiratory rate of 28 breaths per minute. Abdominal examination, cardiovascular and central nervous system examination were within normal limits.

**Figure 1.**
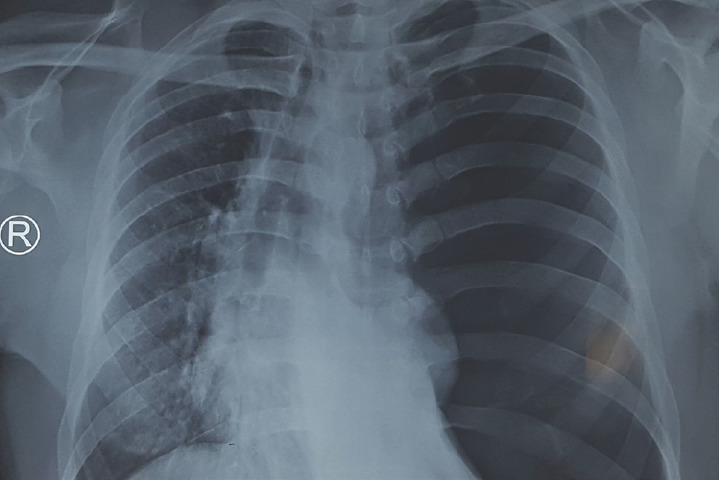
Left sided tension pneumothorax.

His chest x-ray (Figure 1) showed left sided pneumothorax with shifting of trachea and mediasti-num towards right side.

**Figure 2.**
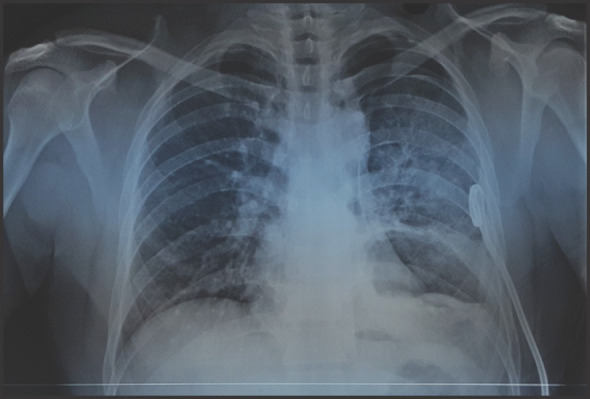
Chest x-ray after pigtail insertion showing lung expansion.

Needle decompression followed by 20Fr pigtail catheter insertion was done in the emergency de-partment itself. Post pigtail catheter insertion chest x-ray (Figure 2) showed re-expansion of affect-ed lung.

His CBC,LFT and RFT were within normal limits and serology for hepatitis B, hepatitis C and HIV were negative. ECG showed no abnormalities. Sputum for AFB was negative and pleural fluid for GenX-pert (TB PCR) was also negative. Pleural fluid was reddish in color, was exudative with LDH of 2933 U/L. Abdominal ultrasound showed only mild fatty liver without any cysts or mass in liver. Hydatid serology (Echinococcus IgG) was positive.

**Figure 3.**
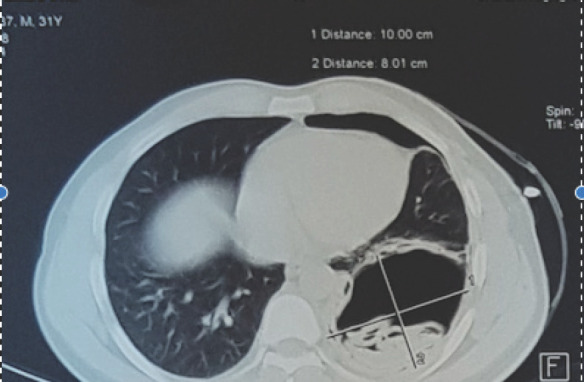
CT chest showing cavitatory lesion with laminated membrane within the cavity.

**Figure 4.**
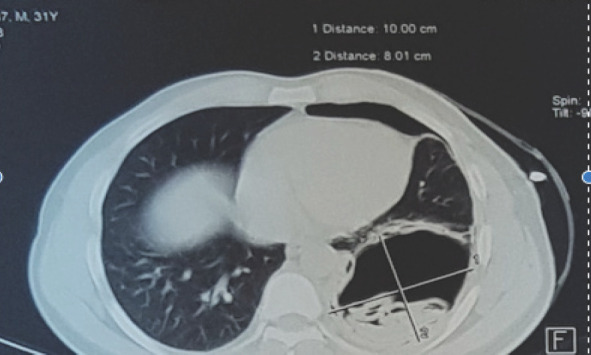
CT chest showing hydatid cyst of the lung.

Post pigtail chest X-ray (Figure 2) showed haziness in the left lower zones, so CT of chest (Figure 3 and Figure 4) was done, which showed thick walled cavitatory lesion in left lower lobe with air fluid level and multiple membrane like structures within the cavity suggestive of ruptured hydatid cyst.

**Figure 5.**
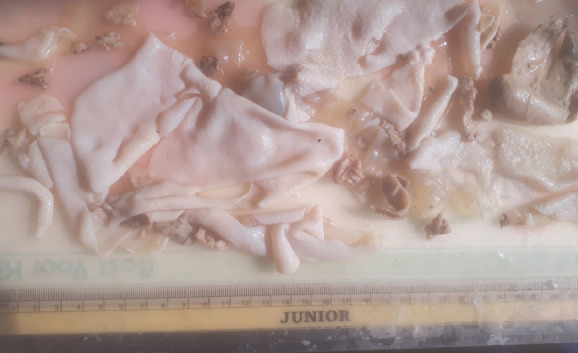
Postsurgeryspecimen after resection.

CTVS consultation was done and the patient underwent surgical resection of the cyst (Figure 5) with pleurodesis.

Postoperative period was uneventful. Tablet albendazole 400mg twice daily in a day was given for 1 months. On long term follow up, the patient remained healthy and there was no recurrence.

## DISCUSSION

Pulmonary hydatid cysts are asymptomatic until they reach a large size and become complicated.^7–10^ A sudden rise in the intrapulmonary pressure, such as coughing, sneezing or an increase in intra-abdominal pressure is the usual risk factor for rupture of the cyst. However rupture may occur spontaneously without any predisposing factor.^11,12^ The occurrences of tension pneumothorax in the rup-ture of the pulmonary hydatid cyst is a rare complication.^11–14^ The signs and symptoms of tension pneumothorax include severe dyspnea, cyanosis, tachycardia, engorgement of jugular vein in the neck and hypotension. The trachea and other components of mediastinum is usually deviated to the contralateral side, increased resonance to percussion on the affected side of the chest and absent breath sounds.^11–13^

In our cases, physical examination of chest and chest radiographs, and CT Chest were of great value in diagnosing the ruptured pulmonary hydatid cyst. The possible mechanism causing tension pneu-mothorax in our case, could be due to the cavity in the lung and the bronchopleural fistula together working as a check-valve mechanism.^10,11^ The current gold standard serology test for echinococcosis detects IgG antibodies to hydatid cyst fluid-derive native or recombinant antigen B subunits. This is performed using ELISA or immunoblot formats.^12^ ELISA test with crude hydatid cyst fluid has a high sensitivity of 95%, however, its specificity is low at 61%.^13^

The treatment of such patients is mainly surgery.^8,12,15^ The aim of surgical intervention in pulmonary hydatid cysts are mainly the removal of the laminated membrane without causing intraoperative contamination and prevention of an intrapulmonary residual cystic space.^7,8,15,16^ For this purpose, varying techniques, such as enucleation, pericystectomy, and simple cystotomy with or without capitonnage of the pericystic space can be chosen in proper conditions during the operation.^8,10,15^ Radical pulmonary resections should be performed when the pulmonary parenchyma around the cyst is destroyed.^7,8,15^

Surgical resection followed by thoracotomy was performed in our case. Post-surgery the patient was comfortable and has no other symptoms during follow up. The possibility of Ruptured pulmonary hydatid cyst as a cause of tension pneumothorax has to be entertained especially in settings with high burden of the disease.

## Consent:

**JNMA Case Report Consent Form** was signed by the patient and the original article is attached with the patient's chart.

## Conflict of Interest

**None.**
